# Ofatumumab in multiple sclerosis: 2-year immune profiling and clinical correlates

**DOI:** 10.1007/s00415-026-14088-2

**Published:** 2026-09-08

**Authors:** Antonio Esposito, Luca Corsaro, Ilaria Delle Cave, Fabiana Capasso, Giuseppe Corsini, Giuseppina Matrone, Vincenza Cerbone, Maria Petracca, Antonio Carotenuto, Roberta Lanzillo, Giulia Scalia, Giuseppe Castaldo, Vincenzo Brescia Morra, Marcello Moccia

**Affiliations:** 1 Department of Neuroscience, Reproductive Science and Odontostomatology, Federico II University of Naples, Naples, Italy; 2 Centre for Advanced Biotechnology (CEINGE), Naples, Italy; 3 Department of Human Neurosciences, Sapienza University, Rome, Italy; 4 Department of Molecular Medicine and Medical Biotechnology, Federico II University of Naples, Naples, Italy

**Keywords:** Multiple sclerosis, Ofatumumab, Lymphocytes, Immune profiling

## Abstract

**Introduction:**

Anti-CD20 monoclonal antibodies, such as ofatumumab, have significantly improved the management of multiple sclerosis (MS). Still, their long-term immunological effects, particularly on non-B-cell populations, remain poorly explored. We characterized longitudinal changes in circulating lymphocyte subsets and explored their clinical correlates in people with MS treated with ofatumumab.

**Methods:**

We conducted an observational cohort study including adults with MS who initiated ofatumumab and had paired baseline and on‑treatment assessments after at least 2 years (*n*=34). Immunophenotyping quantified total lymphocytes and subsets (CD3+, CD3+CD4+, CD3+CD8+, CD19+, CD20+, CD56+, CD27+, CD27+CD3+, CD27+CD19+, CD3+CD20 +). Longitudinal change was modeled with linear mixed‑effects regression (fixed effects: age, sex, follow‑up duration, comorbidities, BMI, previous DMT; random intercept for subject). Annualized percentage change of each laboratory measure was related to relapses, MRI activity, EDSS progression, NEDA‑3, and EDSS improvement using multivariable logistic regression with the same covariates.

**Results:**

Over approximately 2 years of treatment, we observed increased total lymphocytes (Coeff 251.47; 95% CI 29.29–473.64; *p*=0.027), CD3+ (368.79; 183.66–553.91; *p*<0.01), CD3+CD4+ (239.82; 107.32–372.33; *p*<0.01), CD3+CD8+ (132.36; 52.92–211.80; *p*<0.01), CD27+ (275.37; 124.11–426.63; *p*<0.01), and CD27+CD3+ lymphocytes (451.99; 300.40–603.58; *p*<0.01), and decreased CD19+ (− 181.55; −234.40 to −128.70; *p*<0.01), CD20+ (− 181.55; −234.40 to −128.70; *p*<0.01), and CD27+CD19+ lymphocytes (− 1.82; −2.74 to −0.91; *p*<0.01). No significant associations emerged between changes of lymphocytes and relapses, MRI activity, EDSS progression, NEDA‑3, or EDSS improvement.

**Conclusion:**

In this exploratory, descriptive, real-world cohort, ofatumumab was associated with sustained B-cell depletion (CD19+/CD20 +) and parallel increases in broad T-cell compartments. No associations with clinical or MRI outcomes were detected; these negative findings should be interpreted cautiously within the hypothesis-generating design, given the small sample size and low frequency of disease-activity events.

**Supplementary Information:**

The online version contains supplementary material available at https://doi.org/10.1007/s00415-026-14088-2.

## Introduction

Multiple sclerosis (MS) is a chronic inflammatory disease of the central nervous system in which both B and T lymphocytes contribute to tissue injury and disability worsening [1, 2]. Over the last decade, anti‑CD20 monoclonal antibodies have transformed MS care by rapidly suppressing inflammatory disease activity alongside potential long-term effects on disability progression [3]. However, while the clinical outcomes of anti-CD20 therapies are well established, their immunological effects remain partially explored. Emerging evidence suggests that anti-CD20 therapies exert immunological effects beyond B-cell depletion, by affecting T-cell and innate immune cell populations [4, 5]. Ofatumumab, a fully human anti-CD20 antibody delivered as monthly subcutaneous injections, achieved superior control of relapses and MRI activity versus teriflunomide in phase 3 trials, and is now approved for the treatment of relapsing MS [6]. Compared with intravenous anti-CD20 therapies, ofatumumab has distinct pharmacologic features, including primary activity in the peripheral lymphatic compartment, epitope binding and complement-dependent cytotoxicity [7, 8], raising interest in its immune‑modulatory footprint over time [9, 10]. Real-world immunophenotyping suggests that ofatumumab depletes circulating CD20+ B cells [11, 12] along with the small CD3+CD20+ T‑cell subset, while leaving immunoglobulin levels within the normal range [13] and absolute CD4+ and CD8+ lymphocyte counts broadly stable in the short term [14]. Overall, the regulatory balance might improve through increase in T regulatory cells [15], with a shift towards a more anti-inflammatory immune profile [16]. Other studies described early declines in T-cell counts and intermittent lymphopenia [18], particularly in patients who switched from natalizumab or fingolimod. As such, it remains unclear what is the effect of ofatumumab on T lymphocytes, especially in the long term, and potential clinical correlates.

Hereby, we conducted an exploratory and descriptive longitudinal analysis of circulating lymphocyte subpopulations and their clinical correlates in people with MS treated with ofatumumab. Our aims were: (1) to quantify baseline-to-follow-up variation in total lymphocytes and major lymphocyte subsets; and (2) to explore whether changes in these laboratory measures were associated with relapses, MRI activity, disability worsening, or improvement. Analyses of clinical associations were intended to be hypothesis-generating rather than to establish mechanistic pathways or predictive biomarkers.

## Methods

### Study design and population

We performed an observational, real-world cohort study at a tertiary MS center, using prospectively collected data of adults with MS who initiated ofatumumab and had paired baseline and follow‑up assessments with ≥2 years of on‑treatment follow‑up. Ofatumumab was administered as 20 mg subcutaneous injections at Weeks 0, 1, and 2, and monthly thereafter, in accordance with labeling and routine clinical practice [6]. The study was approved by the Campania 3 Ethics Committee, and followed the principles of the Declaration of Helsinki; all participants provided written informed consent.

### Laboratory variables

Peripheral blood was collected in EDTA or heparin tubes and processed using a standardized whole-blood flow-cytometry workflow. For each tube, 50 µL of blood was incubated with the relevant fluorochrome-conjugated monoclonal-antibody mixture for 15 min at 4 °C in the dark. Erythrocytes were lysed with ammonium-chloride solution, samples were centrifuged, and the nucleated-cell pellet was resuspended in 400 µL FACSFlow before acquisition. Samples were acquired on a Navios flow cytometer (Beckman Coulter; 405-, 488-, and 638-nm lasers) and analyzed using Kaluza Analysis software. Lymphocytes were identified on CD45/side-scatter after initial physical-parameter gating. Sequential gates identified CD3+ T cells, CD3+CD4+ and CD3+CD8+ T-cell subsets, CD19+/CD20+ B cells, CD56+CD3− NK cells, CD27+ cells, CD27+CD3+ cells, CD27+CD19+ B cells, and CD3+CD20+ cells [19, 20]. Absolute counts were obtained using the double-platform approach. Representative gates and additional panel details are provided in Supplementary Material 1. Laboratory procedures were conducted under routine quality-control standards, including instrument calibration and UK-NEQAS participation.

### Clinical variables

Baseline variables included age, sex, disease duration, descriptor of disease progression, BMI, comorbidities, previous disease‑modifying therapies (DMTs), and EDSS (acquired by certified examiners). During follow‑up we recorded relapses, MRI activity, EDSS progression, EDSS improvement, and NEDA‑3 (from the combination of relapses, MRI activity, and EDSS progression) [20].

### Statistics

We summarized means ± SD (age, BMI, disease duration, follow‑up duration, total lymphocytes and subsets), medians (range) (EDSS), and *n* (%) (sex, descriptor of disease progression, comorbidities, previous DMTs, relapses, MRI activity, EDSS progression, NEDA‑3, EDSS improvement), as appropriate.

Longitudinal changes of laboratory variables (dependent variable) (aim 1) were modeled via linear mixed‑effects regression with time as the main predictor; fixed effects were age, sex, follow‑up duration, comorbidities, BMI, and previous DMT; a random intercept accounted for repeated measures. Clinical associations (dependent variable) (aim 2) were evaluated using multivariable logistic regression with the annualized percent change of each laboratory measure as the independent variable, and adjusting for the same covariates. In these models, each laboratory measure was evaluated in a separate outcome model; parent and daughter immune populations were therefore not entered simultaneously as competing predictors. Adjustment covariates were selected a priori on clinical grounds, consistently across models.

Results are reported as coefficients (Coeff) or odds ratios (OR) with 95% confidence intervals (95% CI) and *p* values, as appropriate; variables’ distribution was assessed graphically and statistically; two‑sided *p*<0.05 denoted significance; analyses were conducted in Stata 15.0.

## Results

We included 34 people with MS treated with ofatumumab and with available clinical and laboratory data at baseline and follow-up. Demographic and clinical variables are reported in Table 1.

**Table 1:** Demographic and clinical variables

Demographic and clinical variables	*N*=34
Age, years	48.3±11.4
Sex, females	23 (67.6%)
Disease duration, years	14.2±9.6
Descriptor of disease progression	
Relapsing	18 (52.9%)
Progressive	16 (47.1%)
EDSS, median	4.0 (2.0–7.0)
Comorbidities, number	11 (32.3%)
BMI	25.9±3.6
Previous DMTs	
None	9 (26.5%)
Injectables	2 (5.9%)
Orals	18 (52.9%)
Monoclonal antibodies	5 (14.7%)
Follow-up duration, years	2.06±0.50
Relapses, number	2 (5.9%)
MRI activity, number	2 (5.9%)
EDSS progression, number	6 (17.6%)
NEDA-3, number	24 (70.6%)
EDSS improvement, number	7 (20.6%)

**Fig. 1:**
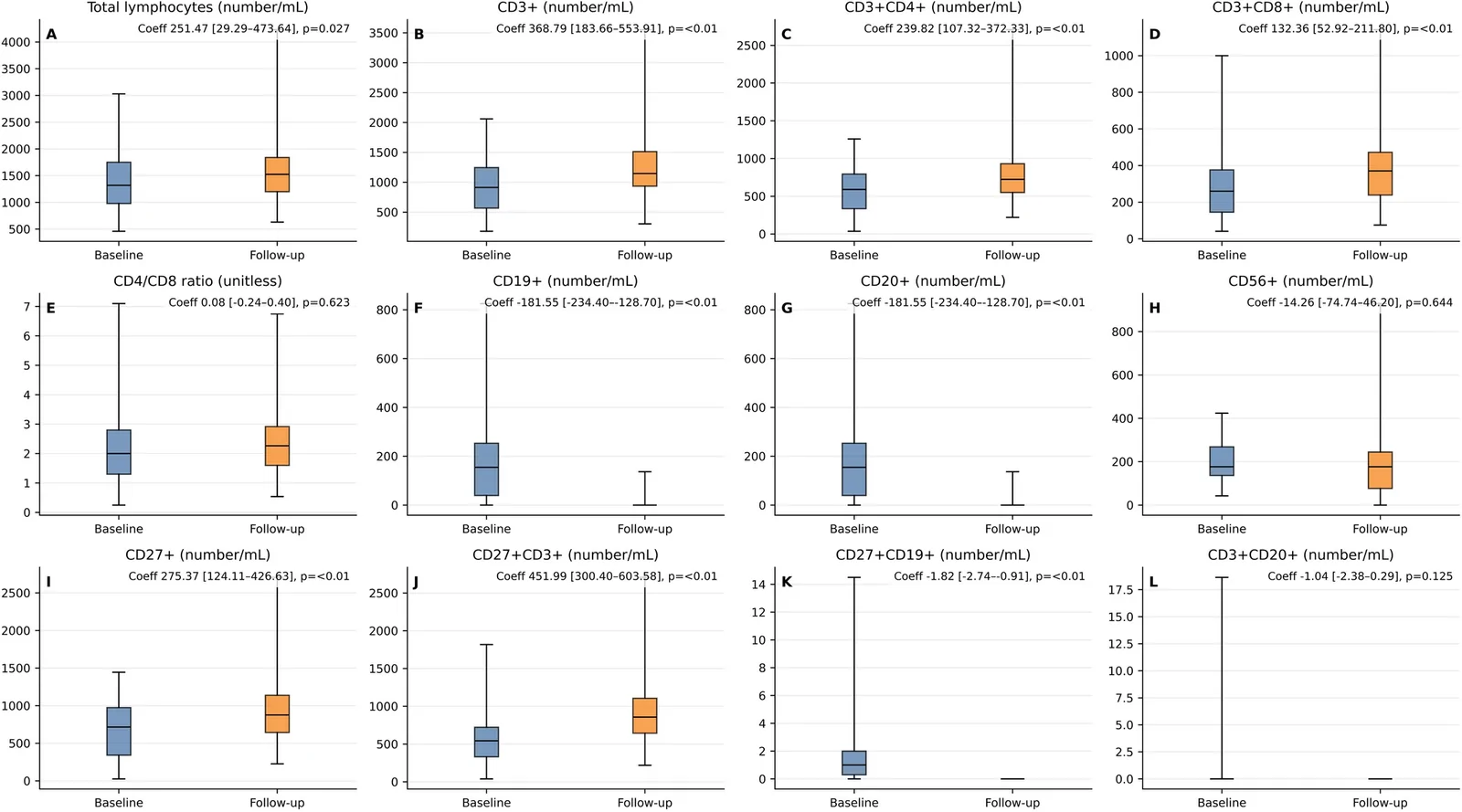
Box‑and‑whisker plots of peripheral lymphocyte subsets at baseline and during treatment with ofatumumab. Box-and-whisker plots represent distribution of different laboratory variables at baseline and follow-up. Statistical estimates are derived from mixed‑effects models

Over the 2-year treatment with ofatumumab, we observed increased levels of total lymphocytes (Coeff=251.47; 95% CI 29.29, 473.64; *p*=0.027), CD3+ (Coeff=368.79; 95% CI 183.66, 553.91; *p*<0.01), CD3+CD4+ (Coeff=239.82; 95% CI 107.32, 372.33; *p*<0.01), CD3+CD8+ (Coeff=132.36; 95% CI 52.92, 211.80; *p*<0.01), CD27+ (Coeff=275.37; 95% CI 124.11, 426.63; *p*<0.01), and CD27+CD3+ lymphocytes (Coeff=451.99; 95% CI 300.40, 603.58; *p* <0.01). By contrast, there were decreased levels of CD19+ (Coeff=−181.55; 95% CI −234.40, −128.70; *p* <0.01), CD20+ (Coeff=−181.55; 95% CI −234.40, −128.70; *p*<0.01), and CD27+CD19+ lymphocytes (Coeff=−1.82; 95% CI −2.74, −0.91; *p*<0.01). Also, there were no statistically significant changes in CD4/CD8 ratio, CD56+, and CD3+CD20+ (Table 2; Fig. 1).

**Table 2:** Variations of total lymphocytes and different lymphocyte subpopulations over time. Statistical estimates are derived from mixed-effects models

	Baseline (number/mL)	Follow-up (number/mL)	Coeff	95% CI		*p* value
				Low	High	
Total lymphocytes	1392.17±616.00	1647.64±727.94	251.47	29.29	473.64	0.027
CD3+	915.77±471.38	1284.56±649.91	368.79	183.66	553.91	<0.01
CD3+CD4+	573.00±327.71	812.83±466.14	239.82	107.32	372.33	<0.01
CD3+CD8+	287.05±189.80	419.41±262.53	132.36	52.92	211.80	<0.01
CD4/CD8 ratio	2.26±1.44	2.37±1.21	0.08	−0.24	0.40	0.623
CD19+	188.99±184.82	7.44±30.34	−181.55	−234.40	−128.70	<0.01
CD20+	188.99±184.82	7.44±20.34	−181.55	−234.40	−128.70	<0.01
CD56+	201.47±97.32	192.29±174.87	−14.26	−74.74	46.20	0.644
CD27+	689.35±424.10	973.22±512.91	275.37	124.11	426.63	<0.01
CD27+CD3+	557.81±386.63	963.19±512.33	451.99	300.40	603.58	<0.01
CD27+CD19+	1.82±3.04	0	−1.82	−2.74	−0.91	<0.01
CD3+CD20+	1.07±4.18	0	−1.04	−2.38	0.29	0.125

We did not find significant associations between longitudinal variations in total lymphocytes or individual lymphocyte subpopulations and relapses, MRI activity, EDSS progression, NEDA-3, or EDSS improvement. Several outcome models could not be reliably estimated or yielded unstable estimates because of sparse clinical/MRI events and/or limited variability in some laboratory measures (including near-complete B-cell depletion). Immune populations were evaluated in separate models rather than entered jointly; therefore, parent and daughter populations were not competing predictors within the same model (Table 3).

**Table 3:** Clinical correlates of annualized changes in lymphocyte measures

	Relapse				MRI activity			
	OR	95% CI low	95% CI high	*p* value	OR	95% CI low	95% CI high	*p* value
Total lymphocytes	n.a				n.a			
CD3+	n.a				n.a			
CD3+CD4+	n.a				0.98	0.95	1.01	0.456
CD3+CD8+	n.a				n.a			
CD4/CD8 ratio	n.a				0.99	0.97	1.01	0.646
CD19+	n.a				0.96	0.80	1.14	0.663
CD20+	n.a				0.96	0.80	1.14	0.663
CD56+	n.a				n.a			
CD27+	n.a				n.a			
CD27+CD3+	n.a				n.a			
CD27+CD19+	n.a				n.a			
CD3+CD20+	n.a				n.a			

	EDSS progression				NEDA-3			
	OR	95%CI		p value	OR	95%CI		p value
		Low	High			Low	High	
Total lymphocytes	1.01	0.96	1.06	0.565	0.96	0.91	1.03	0.322
CD3+	0.99	0.97	1.02	0.980	0.98	0.95	1.01	0.333
CD3+CD4+	0.99	0.95	1.03	0.785	0.98	0.94	1.01	0.255
CD3+CD8+	n.a				0.99	0.96	1.01	0.432
CD4/CD8 ratio	n.a				0.99	0.98	1.01	0.625
CD19+	1.02	0.98	1.05	0.258	1.02	0.98	1.05	0.183
CD20+	1.02	0.98	1.05	0.258	1.02	0.98	1.05	0.183
CD56+	n.a				0.95	0.87	1.03	0.235
CD27+	n.a				0.97	0.93	1.01	0.289
CD27+CD3+	n.a				n.a			
CD27+CD19+	n.a				n.a			
CD3+CD20+	n.a				n.a			

	EDSS improvement			
	OR	95% CI		p value
		Low	High	
Total lymphocytes	1.05	0.98	1.13	0.149
CD3+	1.01	0.99	1.03	0.288
CD3+CD4+	1.00	0.98	1.02	0.568
CD3+CD8+	1.00	0.98	1.01	0.667
CD4/CD8 ratio	n.a			
CD19+	0.97	0.92	1.03	0.367
CD20+	0.97	0.92	1.03	0.367
CD56+	1.01	0.95	1.06	0.718
CD27+	1.00	0.99	1.01	0.256
CD27+CD3+	n.a			
CD27+CD19+	n.a			
CD3+CD20+	n.a			

*n*.*a*. not assessable due to collinearity

## Discussion

The present exploratory and descriptive real-world study investigated longitudinal changes in circulating lymphocyte subpopulations in 34 patients with MS treated with ofatumumab over an approximately 2-year follow-up. We observed sustained depletion of B-cell populations (CD19+, CD20+, CD27+CD19 +), consistent with the known mechanism of action of ofatumumab, together with longitudinal increases in broad T-cell compartments. The biological meaning and clinical relevance of these T-cell changes remain uncertain and should be interpreted as hypothesis-generating.

Our results are in line with previous real‑world, short‑term studies consistently showing rapid and near‑complete peripheral depletion of CD20+ lymphocytes [21, 22] following ofatumumab treatment. Interestingly, previous short-term studies found broadly unchanged absolute CD3+, CD4+, and CD8+ counts [14, 17]. By contrast, we found increased absolute counts of CD3+, CD4+, CD8+, CD27+, and CD27+CD3+ populations. This pattern may indicate broad T-cell compartment reorganization after B-cell depletion [23]. Importantly, the increase in CD27+ and CD27+CD3+ cells should not be necessarily interpreted as evidence of naïve T-cell expansion. CD27 is expressed across both naïve and central memory T-cell compartments and is also present on subsets of NK and NKT cells (CD3+CD56 +). Without additional differentiation markers such as CD45RA/CD45RO and CCR7, the specific CD27-expressing compartment underlying the observed change cannot be resolved [23]. Similarly, the stability of the CD4+/CD8+ ratio suggests that no major shift occurred between these broad T-cell compartments; however, changes in smaller functionally relevant subsets, including Th1, Th17/Th1.17, Tfh, and regulatory T cells, cannot be excluded on the basis of the available panel [17]. A multicenter study conducted on patients who started treatment with ocrelizumab or ofatumumab showed a more significant reduction of B and CD8+ cells in patients treated with ocrelizumab rather than ofatumumab, while total lymphocyte count, CD4+, and NK cells remained similar between two drugs [9]. In keeping with this, a cross‑sectional study during ofatumumab treatment documented depletion of CD20+ T cells, along with improved Th17.1/Treg and Tfh/Tfr ratios, reduced pro‑inflammatory cytokine production, and impaired migration of residual CD20+ T cells across an inflamed in vitro BBB [17]. Discrepancies between studies likely reflect differences in (i) sampling windows (early kinetics vs intermediate vs long‑term immune reorganization); (ii) prior DMT exposures (notably natalizumab or fingolimod) that reshape peripheral pools and trafficking; (iii) measurement approaches (absolute counts vs frequencies; surface‑epitope vs intracellular CD20 staining, the latter avoiding steric hindrance by therapeutic antibodies); and (iv) pharmacologic exposure that might differentiate different anti-CD20 treatments (i.e., subcutaneous ofatumumab vs intravenous ocrelizumab and rituximab) [9, 17, 24]. Taken together, our data, gathered at approximately 2 years, are compatible with a model in which the initial reduction of B cells and CD20+ T cells is followed by qualitative re‑shaping and proportional increase of non‑CD20 T‑cell subsets without overt clinical correlates.

A secondary aim of this study was to explore clinical associations of longitudinal lymphocyte changes. We did not detect associations with relapses, MRI activity, EDSS progression, NEDA-3, or EDSS improvement. However, the low frequency of clinical and radiological disease activity, expected in a cohort receiving a highly effective therapy, together with the small sample size, substantially limited statistical power. Accordingly, the absence of statistically significant associations should not be interpreted as evidence that clinically relevant immunological relationships do not exist. These analyses should therefore be considered exploratory and hypothesis-generating.

Among the main limitations of our study, the relatively small sample size (*n*=34) represents a constraint, which inevitably reduces the statistical power of our findings (especially in relation to the paucity of clinical and MRI outcomes), along with heterogeneity of prior DMTs, lack of functional T-cell readouts (e.g., cytokine profiling, migration assays) and standardized immunoglobulin tracking [14, 17, 25]. Future research should improve phenotyping panels (including Tfh/Tfr and Th17.1), consider intracellular CD20 staining in treated patients, integrate blood biomarker and additional safety outcomes (e.g., infections, vaccine‑response assessments) [9, 10, 17]. More specifically, the available immunophenotyping panel was restricted to broad lymphocyte populations and did not include differentiation markers such as CD45RA/CD45RO and CCR7 or a deeper functional characterization of CD4+ T-cell subsets [23]. Our immune cell characterization did not include the combinations required to identify Th17/Th1.17, regulatory T cells, Tfh/Tfr cells, activation states, cytokine production, or migratory phenotypes [17]. Thus, the present data cannot confirm or refute treatment-related changes described with deeper research panels, and functional interpretation of the observed broad T-cell changes is not possible. In addition, most participants had prior DMT exposure. Although previous DMT was included as a covariate in the adjusted analyses, residual and treatment-specific carry-over effects on immune cell composition and trafficking cannot be excluded. The absence of a parallel ocrelizumab or rituximab comparator group limits direct between-therapy inference, while the lack of standardized intermediate sampling at 6 and 12 months prevents detailed reconstruction of early-to-late immune kinetics.

In conclusion, this exploratory and descriptive study showed sustained B-cell depletion and longitudinal changes in broad T-cell compartments during approximately 2 years of ofatumumab treatment. No associations between immune cell changes and conventional clinical or MRI outcomes were detected. Given the small cohort and low event rate, these negative findings should not be interpreted as excluding clinically relevant immunological associations. Larger prospective studies with repeated longitudinal sampling and deeper immunophenotyping are needed to clarify the kinetics, biological significance, and potential clinical relevance of these immune changes.

## Supplementary Information

Below is the link to the electronic supplementary material.

Supplementary file1 (DOCX 631 KB)

## Declarations

### Data availability

Data are available upon reasonable request to the corresponding author.
