# Supplementary material for: Ofatumumab in multiple sclerosis: 2-year immune profiling and clinical correlates
Source: J Neurol. 2026 Sep 8;273(10):579. doi: 10.1007/s00415-026-14088-2 (PMC13553691; doi:10.1007/s00415-026-14088-2)
Supplement: Supplementary file 1 [file 415_2026_14088_MOESM1_ESM.docx]

Supplementary Material 1. Flow-cytometry panel and representative gating strategy

For the immunophenotypic characterization, fluorochrome-conjugated monoclonal antibodies were used. Each fluorochrome has specific excitation and emission spectra and was selected according to its compatibility with the lasers and optical filters of the flow-cytometry system. The immunophenotyping panel is presented below with antigen target followed by the conjugated fluorochrome and detection channels.

| **Fluorochrome** | FITC | PE | PeCy5 | PE-Cy7 | APC | APC CY7 | HV450 | HV500 |
| --- | --- | --- | --- | --- | --- | --- | --- | --- |
| **Antigen target** | CD20 | CD4 | CD45 | CD56 | CD19 | CD8 | CD3 | CD27 |
| **Excitation laser** | 488 nm (blue) | 488 nm (blue) | 488 nm (blue) | 488 nm (blue) | 638 nm (red) | 638 nm (red) | 405 nm (violet) | 405 nm (violet) |
| **Peak emission (nm)** | ~519 nm | ~578 nm | ~670 nm | ~780 nm | ~660 nm | ~780 nm | ~448 nm | ~510 nm |
| **Emission colour** | Green | Yellow–orange | Violet–red | Dark red | Red | Far red | Blue–violet | Cyan–green |

**Gating strategy**

| **Initial gate** based on physical parameters (**FSC versus SSC**). This step focuses the analysis on the main leukocyte region and excludes obvious debris and extreme events. |  |
| --- | --- |
| **Lymphocyte gate (CD45 versus SSC).** Lymphocytes are identified by bright CD45 expression and low side scatter, reflecting low internal complexity; this separates them from other circulating cells. |  |
| **Total T cells (CD3+ lymphocytes).** |  |
| **Major T-cell subsets (CD3+CD4+ cells).** |  |
| **Major T-cell subsets (CD3+CD8+ cells).** |  |
| **B cells (CD19+CD20+ lymphocytes).** Plot shows a patient with a detectable circulating B-cell population. |  |
| **B cells: CD19+CD20+ lymphocytes.** Plot shows treatment-associated peripheral B-cell depletion in a patient receiving ofatumumab therapy. |  |
| **NK cells (CD56+CD3− lymphocytes).** |  |
| **CD27+CD45+ lymphocytes**. This gate measures total CD27-expressing leukocytes within the lymphocyte region. |  |
| **CD27+CD3+ cells**. CD27 is found on naïve and central-memory T cells and on other subsets. |  |
| **CD27+CD19+ B cells**. Plot shows detectable CD19+ B cells, including a CD27-expressing B-cell fraction. |  |
| **CD27+CD19+ B cells**. Plot shows treatment-associated peripheral CD27+CD19+ cell depletion in a patient receiving ofatumumab therapy. |  |

**Figure 1. Box‑and‑whisker plots of peripheral lymphocyte subsets at baseline and during treatment with ofatumumab***.* Box-and-whisker plots represent distribution of different laboratory variables at baseline and follow-up. Statistical estimates are derived from mixed‑effects models.
